# Clinical Use of a 16S Ribosomal RNA Gene-Based Sanger and/or Next Generation Sequencing Assay to Test Preoperative Synovial Fluid for Periprosthetic Joint Infection Diagnosis

**DOI:** 10.1128/mbio.01322-22

**Published:** 2022-11-10

**Authors:** Laure Flurin, Joseph J. Hemenway, Cody R. Fisher, James J. Vaillant, Marisa Azad, Matthew J. Wolf, Kerryl E. Greenwood-Quaintance, Matthew P. Abdel, Robin Patel

**Affiliations:** a Division of Clinical Microbiology, Department of Laboratory Medicine and Pathology, Mayo Clinic, Rochester, Minnesota, USA; b Department of Intensive Care, University Hospital of Guadeloupe, Pointe-à-Pitre, France; c Division of Public Health, Infectious Diseases and Occupational Medicine, Department of Medicine, Mayo Clinic, Rochester, Minnesota, USA; d Department of Orthopedic Surgery, Mayo Clinic, Rochester, Minnesota, USA; Clinical Center, National Institutes of Health; Brigham and Women's Hospital

**Keywords:** 16S rRNA PCR, metagenomics, next-generation sequencing, periprosthetic joint infection, synovial fluid

## Abstract

Preoperative pathogen identification in patients with periprosthetic joint infection (PJI) is typically limited to synovial fluid culture. Whether sequencing-based approaches are of potential use in identification of pathogens in PJI, and if so which approach is ideal, is incompletely defined. The objective of the study was to analyze the accuracy of a 16S rRNA (rRNA) gene-based PCR followed by Sanger sequencing and/or targeted metagenomic sequencing approach (tMGS) performed on synovial fluid for PJI diagnosis. A retrospective study was conducted, analyzing synovial fluids tested between August 2020 and May 2021 at a single center. Subjects with hip, knee, shoulder, and elbow arthroplasties who had synovial fluid aspirated and clinically subjected to sequence-based testing and conventional culture were studied. A total of 154 subjects were included in the study; 118 had noninfectious arthroplasty failure (NIAF), while 36 had PJI. Clinical sensitivity and specificity for diagnosis of PJI were 69% and 100%, respectively, for the sequencing-based approach and 72% and 100%, respectively, for conventional culture (*P* = 0.74). The combination of both tests was more sensitive (83%) than culture alone (*P* = 0.04). Results of sequencing-based testing led to changes in treatment in four of 36 (11%) PJI subjects. Microbial identification was achieved using Sanger and next generation sequencing in 19 and 6 subjects, respectively. When combined with culture, the described 16S rRNA gene sequencing-based approach increased sensitivity compared to culture alone, suggesting its potential use in the diagnosis of PJI when synovial fluid culture is negative.

## INTRODUCTION

Total joint replacement in the United States is increasing as are numbers of periprosthetic joint infections (PJIs), a devastating complication that occurs following 1 to 2% of primary arthroplasty surgeries (1). Despite recent diagnostic improvements, some PJI cases are difficult to diagnose and remain without pathogen identification (2). Based on Musculoskeletal Infection Society (MSIS) and/or Infectious Diseases Society of America (IDSA) recommendations, definite diagnosis of PJI relies heavily on criteria requiring surgery (e.g., tissue cultures, histopathology, intraoperative purulence); pre-operative diagnosis can be challenging (3, 4).

Preoperatively, clinical examination and inflammatory markers can be indicators of PJI but may be misleading and have poor specificity, especially in patients with underlying inflammatory arthropathies (5). Aspiration of synovial fluid for cell count and differential and, in some cases, markers such as alpha-defensin, can be informative, but these tests do not define the causative organism(s) (6). Cultures of synovial fluid are recommended for preoperative diagnosis of PJI and pathogen identification, though they have been shown to have a relatively low sensitivity across numerous studies (7, 8). Molecular methods such as 16S rRNA (rRNA) gene PCR followed by Sanger sequencing have been shown to have lower sensitivity than synovial fluid cultures (9), possibly due to technical limitations of Sanger sequencing, such as the inability to differentiate polymicrobial samples and a restricted limit of detection. In recent years, next-generation sequencing (NGS) has delivered metagenomic sequencing-based approaches for diagnosis of PJI; these approaches are of particular interest for culture-negative infections (10–12). As a result, NGS was added as a diagnostic tool to the 2018 MSIS criteria for PJI diagnosis (4). However, NGS may be labor-intensive and costly. To facilitate cost-effective application of NGS for bacterial detection, we developed and recently described a targeted metagenomic sequencing-based approach (tMGS) for bacterial detection and identification in normally sterile tissues and body fluids that uses Sanger sequencing and/or NGS after 16S rRNA gene PCR amplification, with sequencing type dependent on the cycle threshold (Ct) value obtained (13). At high Ct values, the assay is reported as negative without sequencing. This assay has been available as a routine test ordered at the discretion of clinicians at our institution. The objective of this retrospective study was to assess sensitivity of this sequencing-based approach applied to synovial fluid for PJI diagnosis, compared to conventional culture.

## RESULTS

### Subjects.

**FIG 1:**
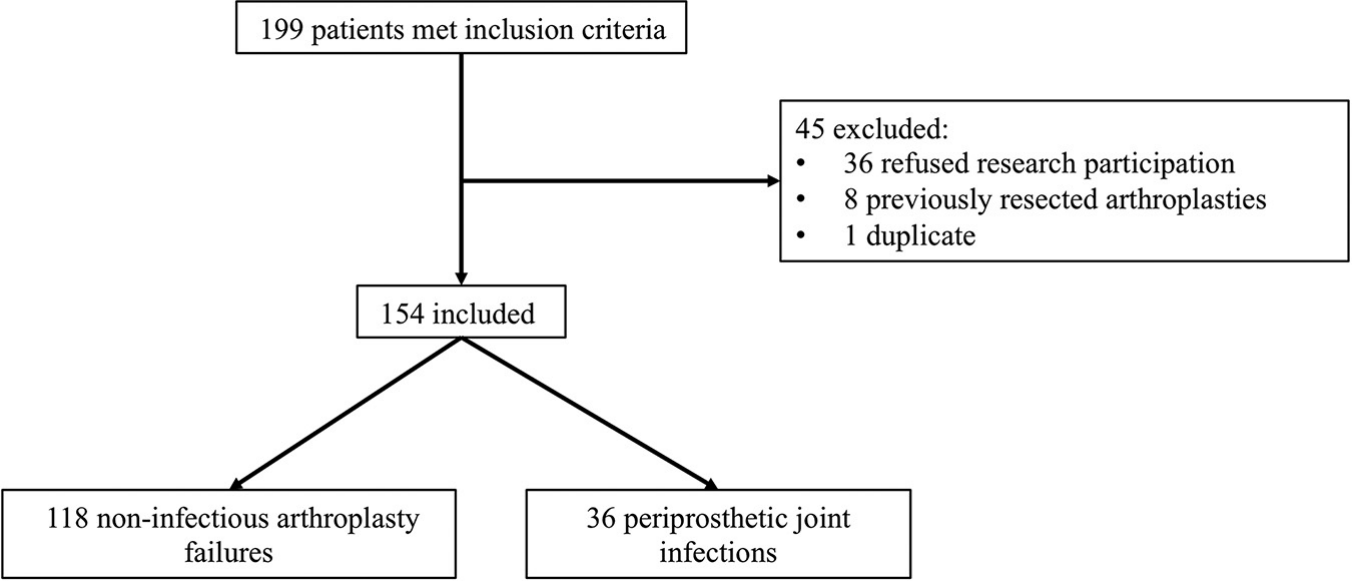
Flow chart showing numbers of subjects studied.

One hundred and ninety-nine subjects met inclusion criteria: 36 were excluded because they refused research participation; 8 because they had a previously resected implant; and 1 because of being a duplicate sample—in total, 154 were included, of which 36 were classified as having PJI and 118 non-infectious arthroplasty failure (NIAF) (Fig. 1). Median age was 66.5 years and 65.5 years in the NIAF and PJI groups, respectively, with most subjects in each group having a knee arthroplasty. In the NIAF group, 20 (17%) had received antibiotic therapy in the 4 weeks before synovial fluid sampling, compared to 23 (64%) in the PJI group (*P* < 0.0001, Table 1).

**TABLE 1:** Patient characteristics^*a*^

Patient characteristics	Noninfectious arthroplasty failure *n* = 118	Periprosthetic joint infection *n* = 36	*P* value
Age, yrs, median [IQR]	67 [61−75]	66 [58−73]	0.450
Female, n (%)	62 (53)	15 (42)	0.341
Arthroplasty type			
Hip, n (%)	35 (30)	8 (22)	0.524
Knee, n (%)	69 (58)	25 (69)	0.329
Shoulder, n (%)	13 (11)	3 (8)	0.765
Elbow, n (%)	1 (0.9)	0 (-)	-
Body mass index, kg/m^2^, median [IQR]	31 [26−35]	30 [26−37]	0.610
Type 2 diabetes, n (%)	20 (17)	7 (19)	0.803
Long-term immunosuppressive therapy, n (%)	10 (8)	4 (11)	0.741
Antibiotic therapy, ≤4 wks before sampling, n (%)	20 (17)	23 (64)	<0.0001
Patients who underwent surgical treatment, n (%)	49 (42)	28 (78)	0.0002
Clinical findings			
Pain, n (%)	112 (95)	33 (92)	0.437
Fever, n (%)	2 (2)	4 (11)	0.027
Swelling, n (%)	23 (19)	11 (32)	0.173
Erythema, n (%)	4 (3)	5 (14)	0.033
Wound dehiscence, n (%)	1 (1)	5 (83)	0.0028
Sinus tract, n (%)	0 (-)	0 (-)	-
C-reactive protein, mg/L, median [IQR]	4.0 [3.0−10.8]	35.7 [15.4−54.4]	<0.0001
Erythrocyte sedimentation rate, mm/h, median [IQR]	10 [5−28]	39 [25−90]	<0.0001
Blood leukocytes, 10^9^/L, median [IQR]	7.1 [5.6−8.5]	8.9 [7.2−10.2]	0.0016
Synovial fluid analysis	*n* = 110	*n* = 35	
Nucleated cell count/μL, median [IQR]	657 [221.5−1991]	26675 [4867−56336]	<0.0001
Neutrophil percentage, median [IQR]	22 [4−56]	89 [83−96]	<0.0001
Positive alpha-defensin test, (NIAF *n* = 57; PJI *n* = 15) n (%)	3 (5)	12 (80)	<0.0001
Synovial fluid microbiology testing	*n* = 118	*n* = 36	
Synovial fluid culture positive, n (%)	0 (−)	26 (72)	-
t MGS positive, n (%)	0 (−)	25 (69)	-
Ct value <32 cycles, n (%)	0	21 (58)	
Positive by Sanger sequencing	0	19/21	-
Positive by NGS/no. sent to NGS	0/0	2/2	-
Ct value ≥32-34 cycles	7 (6)	1 (28)	-
Positive by NGS/no. sent to NGS	0/7	1/1	-
Ct value >34 cycles	111 (94)	14 (39)	-
Positive by NGS/no. sent to NGS	0/0	3/3	-
Impact of tMGS on clinical management, n (%)	0 (−)	4 (11%)	

*^a^*IQR, interquartile range; Ct, crossing threshold; NGS, next-generation sequencing; tMGS, targeted metagenomic sequencing-based approach.

### tMGS results.

In the NIAF group, no synovial fluid sample had a Ct value below 32; 7 (0.6%) had Ct values between 32 and 34 and were sent to NGS, with none ultimately reported as positive; and 111 had Ct values above 34 and were reported negative without sequencing being performed (Table 1).

In the PJI group, 21 (58) had Ct values below 32, among which 19 were positive by Sanger sequencing and 2 by NGS (after being uninterpretable by Sanger sequencing). A single sample had a Ct value between 32 and 34; it resulted in a positive result with NGS. Three synovial fluid samples had Ct values above 34 with melting temperature peaks ≥0.4, all of which yielded positive results with NGS. All six samples sent to NGS were reported as positive and 19 of 21 sent to Sanger sequencing were reported as positive based on Sanger sequencing, for a total of 25 samples positive with the assay (Table 1).

### Comparison to synovial fluid culture.

**FIG 2:**
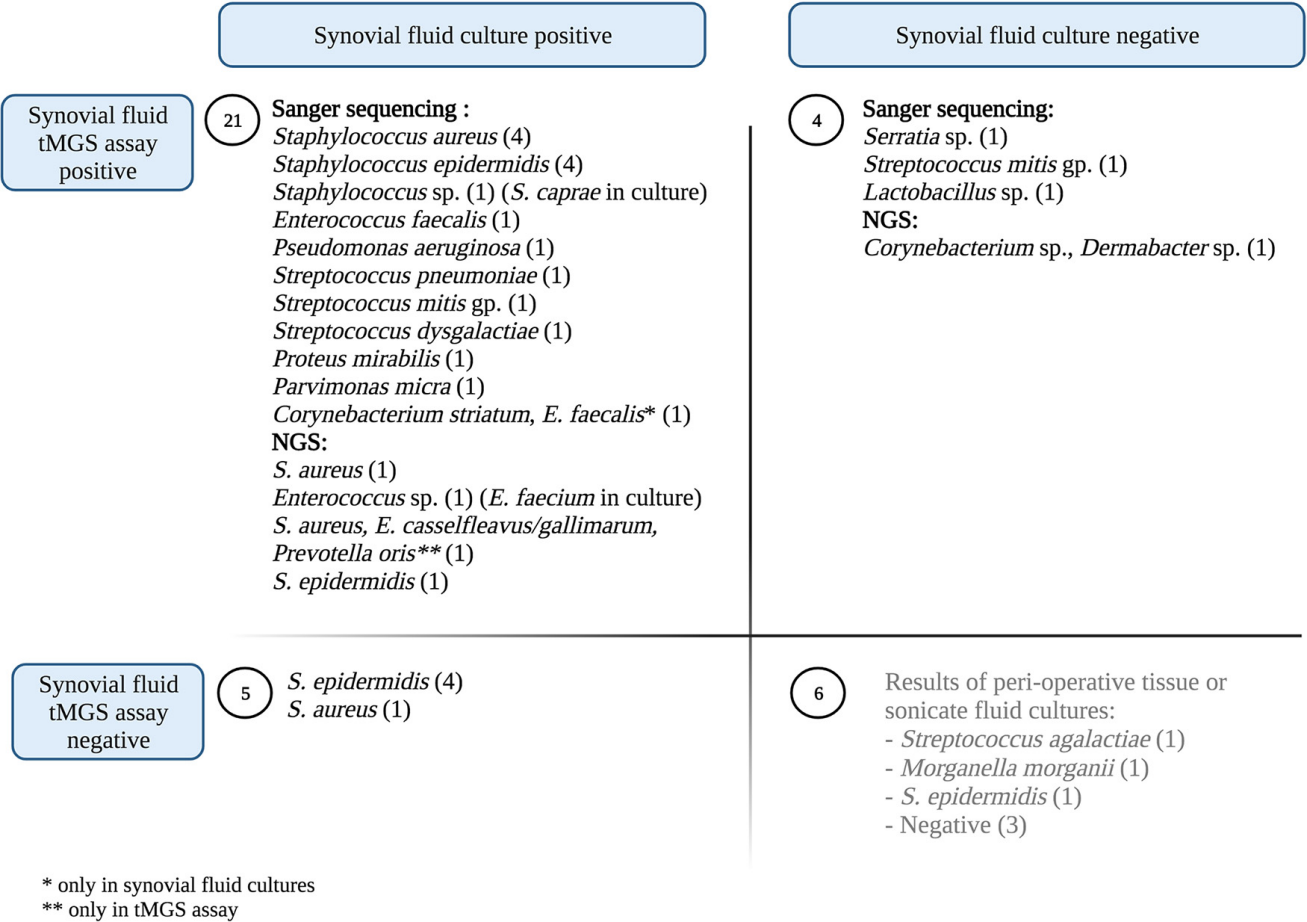
Results of synovial fluid cultures and testing with the targeted metagenomic sequencing-based assay (tMGS) in periprosthetic joint infection and noninfectious arthroplasty failure. NGS, next-generation sequencing. Created with Biorender.com.

Clinical sensitivity of synovial fluid culture was 72% compared to 69% for the sequencing-based approach (*P* = 0.74) (Table 2). Four Staphylococcus epidermidis and one Staphylococcus aureus culture-based detections were missed by tMGS. However, tMGS was positive in four culture-negative cases, detecting *Serratia* species most closely related to Serratia marcescens, Streptococcus mitis group, *Lactobacillus* species, and one polymicrobial sample comprised of *Corynebacterium* and *Dermobacter* species (Fig. 2).

**TABLE 2:** Clinical sensitivity, specificity, and positive and negative predictive values of synovial fluid culture and the targeted metagenomic sequencing-based approach (tMGS) in 36 periprosthetic joint infection and 118 non-infectious arthroplasty failure cases

	Sensitivity %, [95% CI]	Specificity %, [95% CI^*b*^]	Positive predictive value %, [95% CI]	Negative predictive value %, [95% CI]
Synovial fluid culture	72 [56−84]	100 [97−100]	100 [87−100]	92 [86−96]
Synovial fluid tMGS	69 [53−82]	100 [97−100]	100 [87−100]	91 [85−95]
Synovial fluid, 16S rRNA PCR and interpretable Sanger sequencing results	53 [37−68]	100 [97−100]	100 [83−100]	87 [81−92]
Synovial fluid culture and/or tMGS	83^*a*^ [68−92]	100 [97−100]	100 [89−00]	95 [90−98]

*^a^P* < 0.05 compared to synovial fluid culture alone.

*^b^*CI: confidence interval.

Considering the complementary results of both tests, sensitivity of synovial fluid culture when combined with tMGS was 83%, higher than culture alone (*P* = 0.04). In all cases, the positive predictive value was 100%; that is, positive results correlated with PJI in every case. Negative predictive values were 92%, 91%, and 95% for synovial fluid culture, tMGS, and both combined, respectively (Table 2). In the subgroup of 23 PJI subjects who had received antibiotics in the 4 weeks before sampling, sensitivity was 70% for synovial fluid cultures and 65% for tMGS (*P* = 0.71). Among the six subjects with PJI with negative culture and tMGS results, five underwent revision surgery after synovial fluid aspiration, three of whom had positive peri-operative cultures from either sonicate fluid and/or tissue, identifying Streptococcus agalactiae (in tissue and sonicate fluid cultures), Morganella morganii (in tissue cultures; sonicate cultures were not performed), and S. epidermidis (in sonicate fluid cultures only).

### Value of adding NGS to Sanger sequencing.

The sensitivity of 16S rRNA PCR followed by Sanger sequencing alone (interpretable sequencing results) was 53% (*P* = 0.052 compared to synovial fluid culture) (Table 2). The additional yield of positive results with NGS compared to Sanger sequencing alone was 32%.

### Impact on clinical decision making.

Of the 25 positive tMGS tests, results had an impact on clinical management of four subjects (16%), including 11% in the PJI group. For one subject with a tMGS finding of *Serratia* species and negative synovial fluid culture, surgical revision (two-stage) and antibiotic treatment was employed. For one subject with tMGS detection of S. mitis species group, antimicrobial treatment was deescalated from vancomycin and ceftriaxone to ceftriaxone alone. For one polymicrobial infection detected by synovial fluid culture (S. aureus and Enterococcus faecalis), tMGS additionally identified Prevotella oris for which anaerobic coverage was added. In one subject with a negative synovial fluid culture, the tMGS assay identified *Corynebacterium* and *Dermabacter* species, for which management was escalated to revision surgery, followed by 3 months of daptomycin and cefepime and ultimately chronic suppression with doxycycline and meropenem. Later, tMGS testing of periprosthetic tissue confirmed the same microorganisms.

## DISCUSSION

This study reports the clinical performance of a 16S rRNA gene sequencing-based assay ordered routinely to determine infection associated with prosthetic joints, compared to conventional cultures. The assay was designed to fill the gaps of Sanger sequencing alone by using NGS when Sanger sequencing failed or was likely to do so. In this way, performance of NGS, which is costly and labor-intensive, was limited to situations where it was likely to be most helpful.

In this study of synovial fluid, and 16S rRNA gene-based PCR, 6 samples were reported as positive based on NGS, and 19 based on Sanger sequencing (Fig. 2). In total, the sequencing-based assay detected five potential pathogens in four culture-negative synovial fluids and detected one additional anaerobe in a polymicrobial sample. Nevertheless, the assay’s clinical sensitivity was not superior to that of synovial fluid culture for diagnosis of PJI, as it missed five microorganisms in culture positive samples, and one in a polymicrobial sample. However, the two tests were complementary, with a clinical sensitivity of 83% when combined, higher than that of culture alone. Antibiotic use prior to aspiration did not appear to affect sensitivity of culture or the sequencing-based assay. However, sample size was small, and a larger cohort would be required to further investigate this subject. The role of the described sequencing-based assay in the management of patients with suspicion of PJI is therefore likely as a complement to culture rather than a replacement. An impact on clinical management was found in four out of 25 subjects with positive tMGS testing (16%).

Studies on the use of 16S rRNA PCR followed by Sanger sequencing in synovial fluid are sparse; this method has been evaluated in sonicate fluid and shown to have comparable clinical sensitivity to sonicate fluid culture alone (14). A study in 40 PJI patients evaluating the use of 16S rRNA PCR followed by Sanger sequencing on multiple sample types (synovial fluid, sonicate fluid and periprosthetic tissues) showed similar results, with clinical sensitivities of 67% for PCR and 76% for culture (15). Multiplex PCR (Unyvero, Curetis, Holzgerlingen, Germany), which has the advantage of quicker results and is more easily performed, showed a sensitivity of 60% in a study of 77 PJI cases by Morgenstern et al., with difficulty detecting Cutibacterium acnes and S. epidermidis (16). Further evaluation, using the recently FDA cleared/approved BioFire Joint Infection Panel (bioMérieux) should be performed. Another metagenomic sequencing approach, shotgun metagenomic sequencing (sMGS) has shown mixed results in synovial fluid, with reported sensitivities higher than 90% in some studies (17, 18), and comparable sensitivities to culture in others (19). Results of sMGS of synovial fluid reported by Ivy et al. in a study of 107 PJI cases reported similar sensitivity to tMGS, with a clinical sensitivity of 67%, compared to 77% for culture (10). Interestingly, nine out of 14 pathogens missed by sMGS were S. epidermidis, compared to four out of five missed by tMGS in this study. This is likely because it is hard to interpret low levels of S. epidermidis detection in NGS data since it can be present as assay background (20).

Prior to the introduction of NGS into the described assay, it was exclusively based on Sanger sequencing, and only samples with Ct values equal or below 32 cycles were sent to sequencing, as described in (21). Considering the higher sensitivity of NGS, the threshold for sequencing was increased to 34 cycles. This strategy minimized numbers of false positives and labor, cost and turnaround time of the assay (13). As shown here, for synovial fluid, most positive results were obtained using Sanger sequencing (19/25, 53% sensitivity). Additional positive results may have been obtained in the subgroup of samples with Ct values above 34 cycles not submitted to sequencing as, of the three samples with Ct values >34 cycles that underwent NGS, all yielded microbial detections. Therefore, reassessment of the Ct threshold could be considered for synovial fluid, and may theoretically increase assay sensitivity.

Limitations of this study include the small sample size and its retrospective design. However, as the cohort was recruited prospectively, the proportion of NIAF (77%) and PJI (23%) subjects is a reflection of real-life testing and aligns with the literature where infection represents approximately 25% of arthroplasty failures (22). PJI was defined using IDSA criteria; in cases not clearly defined, review by three clinicians specialized in orthopedic infectious diseases was done. This study does not address the value of synovial fluid testing prior to reimplantation (second stage of two-stage revision arthroplasty surgery).

In summary, in this cohort of 154 patients, including 36 with PJI, testing of synovial fluid with the described tMGS approach had similar diagnostic yield to culture for PJI diagnosis. However, the sequencing-based assay identified four potential pathogens in 10 culture-negative PJIs and had a clinical impact on four patients. Adding NGS to a Sanger sequencing-based 16S rRNA gene-based approach increased the positivity rate, without affecting specificity. The described sequencing-based approach may be useful in synovial fluid culture-negative PJI and should be further studied in a larger cohort.

## MATERIALS AND METHODS

### Patient cohort and characteristics.

This retrospective study was conducted between August 2020 and May 2021 at a single medical center. Patients with total hip, knee, shoulder, and elbow arthroplasties who had their joints aspirated preoperatively for clinical suspicion of PJI and synovial fluid submitted for both tMGS and conventional cultures were included. Exclusion criteria included refusal to participate in research, duplicate testing on the same subject, and previously resected arthroplasties. The decision to send the sample for tMGS was at the discretion of the clinical team. Each subject’s electronic medical record was reviewed to document patient characteristics and medical history, as well as whether antibiotics had been taken within the 4 weeks leading up to aspiration. The onset of symptoms was calculated as the time elapsed between the last arthroplasty surgery and the first clinical finding that led to consultation. In addition, clinical details on the day of aspiration (fever, pain, swelling, erythema, wound drainage, sinus tract), laboratory findings, and radiographic results were collected. Results of synovial fluid analysis were also gathered, including total nucleated cell count, neutrophil percentage, and alpha-defensin, if available. If the patient underwent revision surgery after aspiration, the type of revision surgery and results of tissue and/or sonicate fluid cultures from the surgery were recorded. Classification of PJI and noninfectious arthroplasty failure (NIAF) were assessed based on IDSA criteria by a medical doctor (3). If classification was uncertain, the case was independently reviewed by three medical doctors specialized in orthopedic infections and a majority decision assigned. Impact of sequencing-based testing on clinical decision making was considered present if specifically noted in the subject’s medical record by their provider in association with escalation or deescalation of antimicrobial treatment or leading to a specific surgical plan.

### Synovial fluid cultures.

Culture methods used were dependent on the volume of synovial fluid received. If ≥2 mL was received (recommended volume), 1 mL was inoculated into each of Bactec aerobic and anaerobic bottles and the bottles incubated on a Bactec FX Instrument (BD Diagnostic Systems, NJ) for 14 days. For volumes <2 mL, for aerobic cultures, synovial fluid was streaked on TSA II with 5% sheep blood agar and chocolate II agar plates (BD Diagnostic Systems, NJ) and incubated at 37°C with 5% to 7% CO_2_ for 5 days, and inoculated in thioglycolate broth (BD Diagnostic Systems, NJ) and incubated for 5 days at 37°C. If the broth appeared turbid, it was subcultured. For anaerobic cultures, synovial fluid was inoculated on TSA II with 5% sheep blood anaerobic agar and CDC anaerobic blood agar plates (BD Diagnostic Systems) as well as into Remel enriched thioglycolate medium (Thermo Fisher Scientific, Waltham, MA) and incubated anaerobically for 14 days. Positive blood culture bottles, and/or broths were subcultured; organisms were identified with matrix assisted laser desorption ionization-time of flight (MALDI-TOF) mass spectrometry. Positive cultures were defined as organism growth, regardless of quantity or medium.

### Targeted metagenomic sequencing.

A 100 μL sample of synovial fluid was lysed with 20 μL of 0.1 mm silica/zirconium beads, 160 μL proteinase K buffer (PKB), and 20 μL of proteinase K. Lysis tubes were spun down, incubated/shaken at 60°C/300 rpm for 1 h, and then again at 100°C/2000 rpm for 5 min. Lysis tubes were cooled for 5 min at room temperature and then centrifuged for 30 s at 10,000 × *g*. After, 270 μL of lysed sample was added to 2 mL of prewarmed (40°C) NUCLISENS easyMAG lysis buffer (bioMérieux, Marcy-l’Étoile, France) in a disposable NUCLISENS easyMAG cartridge and loaded on a NucliSENS easyMAG or EMAG (bioMérieux). 20 μL of eluate was obtained.

Five μL of extracted DNA was mixed with 15 μL of mastermix containing primers targeting the V1 through V3 regions of the 16S rRNA gene, before loading on a LightCycler 480II (Roche Diagnostics, Risch-Rotkreuz, Switzerland), as previously described (23). SYBR green DNA detection was used to determine Ct values. Samples with Ct values <32 cycles were sent to Sanger sequencing. Samples with Ct values ≥32 and ≤34 or <32 with Sanger sequencing yielding a negative or poor-quality result, or mixed chromatograms, were sent to NGS. Samples with Ct values >34 cycles were not further analyzed and returned negative, except if a well-defined (≥0.4) melting temperature (*T_m_*) peak was observed, in which case they were sent to NGS.

An applied Biosystems 3500xl instrument (Thermo Fisher Scientific) was used for Sanger sequencing and an Illumina MiSeq with a 500 cycle (2 × 250 paired-end read) v2 nano kit for NGS. Sequencing results were processed with RipSeq NGS software (Pathogenomix, CA), as previously described (13).

### Statistics.

Qualitative values were compared using *t* test or Fisher exact test, as appropriate. Quantitative values were tested for normality using the Kolmogorov-Smirnov test and compared using Mann-Whitney or unpaired t-tests, as appropriate. Clinical sensitivities, specificities, and negative and positive predictive values were calculated using 2 × 2 contingency tables, based on infection status (PJI versus NIAF). 95% confidence intervals were calculated as exact binomial confidence intervals. To compare clinical performance, a McNemar's test of paired proportions was performed. *P* values <0.05 were considered statistically significant.

### Ethics.

This study was reviewed and approved by the Mayo Clinic Institutional Review Board (IRB 20-012373).
